# Omitted Medications Among Hospitalized Older Adults and Persons with Dementia Using MIMIC-IV

**DOI:** 10.1093/geroni/igaf122.3987

**Published:** 2025-12-31

**Authors:** Ellen Munsterman, Jiyoun Song, Marie Boltz, Pamela Cacchione

**Affiliations:** University of Pennsylvania School of Nursing, Philadelphia, Pennsylvania, United States; University of Pennsylvania School of Nursing, Philadelphia, Pennsylvania, United States; Pennsylvania State University, University Park, Pennsylvania, United States; University of Pennsylvania, Philadelphia, Pennsylvania, United States

## Abstract

Omitted medication occurs when a prescribed drug is not administered. Hospitalized persons with dementia (PWD) may experience omitted medications due to delirium, lack of routine/familiarity, and/or lack of dementia-specific training among clinicians. The impact of omitted medications ranges from negligible to fatal, but little research has been done on omitted medications focused on hospitalized persons with dementia. This study aimed to examine the association between having a dementia diagnosis and omitted medications and explore factors that may influence this relationship. We utilized the MIMIC-IV-Hosp dataset, de-identified EHR data from adults admitted to Beth Israel Deaconess Medical Center from 2008-2019. Our sample included admission-level data for adults aged >60 with hospital stays >48 hours. PWD were identified using ICD diagnosis codes. Omitted medications were identified based on event documentation in the medication record. Preliminary findings: Among 95,421 admissions, 10,921 (11.4%) were PWD. In unadjusted comparisons, PWD had a 2.65% higher proportion of omitted medications (p < 0.001). After adjusting for age and length of stay (LOS), PWD had a 1.95% higher proportion of omitted medications (p < 0.001). While statistically significant, the effect size was small, and the model explained only 2.3% of the variance. Among PWD, delirium was independently associated with a higher proportion of omitted medications (β = 2.61, p < 0.001), adjusting for age and LOS. This model explained 3.9% of the variance in omitted medications among PWD. Further research is warranted to identify types of medications most affected by omissions among PWD and to clarify the underlying mechanisms.

